# Cancer’s Metabolic Hijack: The Under-Recognized Hematologic Emergency Related to the Warburg Effect

**DOI:** 10.7759/cureus.77869

**Published:** 2025-01-23

**Authors:** Weiying Li, Seema Jaga, Shuva Shah, Martin Cearras

**Affiliations:** 1 Internal Medicine, AdventHealth Orlando, Orlando, USA; 2 Critical Care Medicine, AdventHealth Orlando, Orlando, USA

**Keywords:** aerobic glycolysis, lactic acidosis, lymphoma, refractory hypoglycemia, warburg effect

## Abstract

The Warburg effect, also referred to as aerobic glycolysis, describes the dysregulated energy metabolism in cancer cells with rewired energy generation, which is the hallmark of metabolic shift in cancer. It is a sign of “acute cancer” and is correlated with high malignancy burden and potential aggressive leukemia transformation in lymphoma. We present a case of T-cell lymphoma that developed into life-threatening clinical syndrome driven by the Warburg effect manifesting as severe type B lactic acidosis and refractory hypoglycemia.

A 39-year-old female with a history of NK T-cell lymphoma and airway narrowing secondary to bulky cervical lymphadenopathy presented with fever after recent chemotherapy. She had developed a high fever on day eight of her second cycle of chemotherapy. On presentation, she reported neck pain and generalized fatigue; her peak temperature was 38.9 °C (101.5 °F), but she was hemodynamically stable. Physical examination revealed bulky axillary lymphadenopathy. Laboratory findings revealed pancytopenia, lactic acid of 4.9 mmol/L, and glucose of 57 mg/dL. She received a blood transfusion and empiric antibiotics. Interestingly, she developed refractory hypoglycemia that did not respond to standard treatment. Despite being on an infusion of dextrose 10% at the rate of 100 ml/hour and repeat pushes of dextrose 50%, she continued to experience drops in blood glucose levels into the 40s six times. Extensive workup ruled out active infection, insulinoma, and factitious use of anti-diabetic drugs. She was transferred to the ICU due to worsening lactic acidosis and refractory hypoglycemia; her lactic acid peaked at 14.40 mmol/L. At first glance, it was puzzling as to why she was hypoglycemic despite being switched to dextrose 20% infusion at 150 ml/hour in the ICU. Her blood glucose dropped below 70 mg/dL multiple times and required repeated pushes of dextrose 50%, but her blood glucose still kept dropping.

Our review of the literature revealed that this classic clinical syndrome is driven by the Warburg effect - an ominous sign of aggressive leukemia transformation and high tumor burden. The patient's clinical status rapidly deteriorated and she was intubated for impending respiratory distress, complicated by life-threatening bleeding due to disseminated intravascular coagulation (DIC) and malignant high fever. In light of the extremely poor prognosis, her family decided to withdraw aggressive care, and the patient expired the same night after the withdrawal of care. The classic clinical syndrome driven by the Warburg effect can be an early sign of impending clinical decompensation, and early recognition enables clinicians to appreciate the grim nature of the illness and initiate prompt chemotherapy to improve outcomes. As a hallmark of tumor cells, the Warburg effect has also become a promising therapeutic target in cancer treatment.

## Introduction

The Warburg effect is a well-defined hematologic emergency and it is associated with very high mortality rates [1]. Rapidly proliferating cancer cells convert tremendous amounts of glucose into lactate swiftly by switching from oxidative respiration to fermentation, which confers a growth advantage to cancer cells but also results in hypoglycemia and type B lactic acidosis [2]. We present a case of T-cell lymphoma that developed into a life-threatening clinical syndrome driven by the Warburg effect manifesting as severe type B lactic acidosis and refractory hypoglycemia.

## Case presentation

A 39-year-old Haitian Creole-speaking female with a history of NK T-cell lymphoma and bulky axillary lymphadenopathy presented to the hospital with a fever after recent chemotherapy. She had been diagnosed with lymphoma in November 2023 in the U.S. Virgin Islands. The biopsy had shown “large cell lymphoma” and caseating granuloma, and she had received RIPE (rifampin, isoniazid, pyrazinamide, ethambutol) therapy for suspected tuberculosis but the therapy had only lasted for 1.5 weeks. She had then been lost to follow-up and relocated to the United States.

She presented to the hospital complaining of fever, night sweats, and unintentional weight loss. Extensive workup for infection and tuberculosis was negative. She received an excisional lymph node biopsy in December 2023, which revealed high-grade mature T/NK cell lymphoma with CD30 partially positive, proliferating index of more than 90%, HTLV1 DNA (+). She received one cycle of CHOEP (cyclophosphamide, hydroxydaunorubicin, vincristine, etoposide, and prednisone) regimen in December. Two months later, her oncologist transitioned her to the SMILE (methotrexate, leucovorin, dexamethasone, etoposide, mesna, ifosfamide, filgrastim) regimen and the first cycle was given in February. She was scheduled to receive peg-asparaginase as an outpatient; however, she did not show up for infusion and also missed her appointment for the second cycle of SMILE therapy.

**Figure 1:**
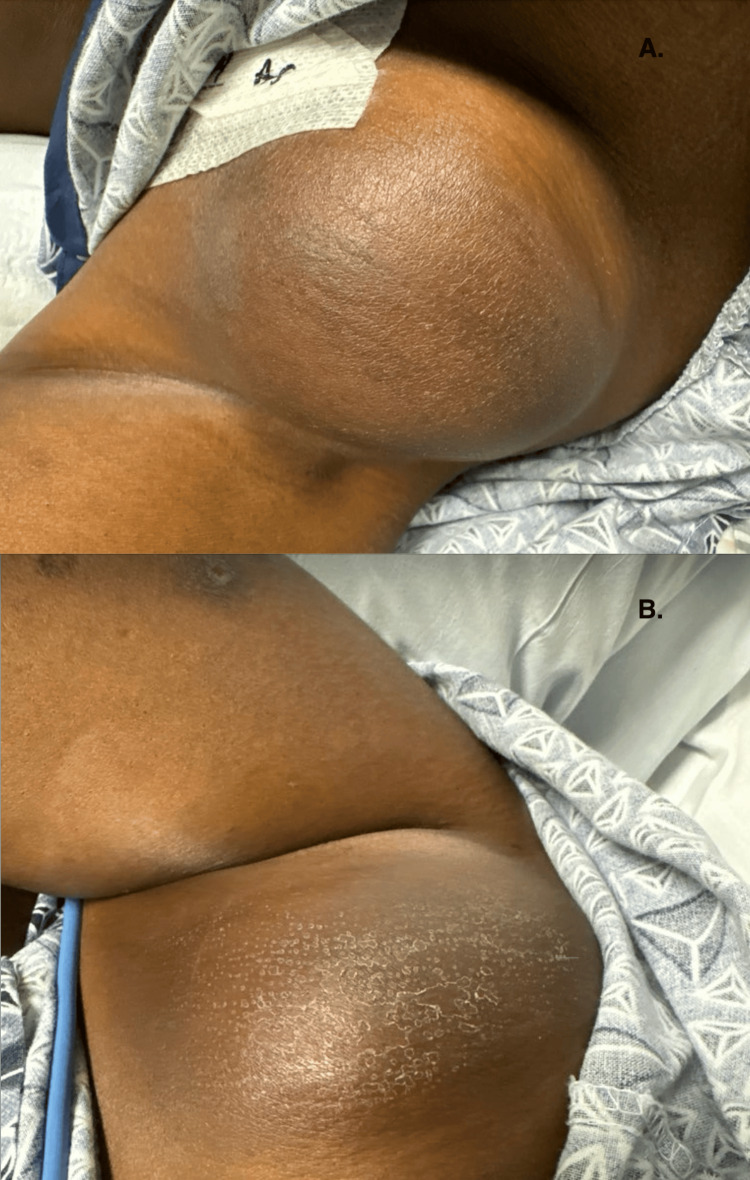
Bilateral axillary lymphadenopathy A: Left axillary lymphadenopathy. B: Right axillary lymphadenopathy

She presented to the hospital two months later in April due to neck pain; a CT revealed narrowing of pharynx secondary to bilateral bulky cervical adenopathy measuring 6.7 x 5.4 cm (previously 5.6 x 4.3 cm) with necrosis and mass effect on trachea secondary to enlarged palatine tonsils. She then received the second cycle of SMILE therapy and a dose infusion of peg-asparaginase. One month later, she presented to the hospital for shortness of breath due to a mass effect, and she received radiation therapy. She was started on the PGEMOX regime (gemcitabine, oxaliplatin, peg-asparaginase) during her hospital stay in May. She received the second cycle of PGEMOX chemotherapy five weeks later. On day eight of her second cycle, she was sent to the hospital by a nurse at the infusion center due to a high fever. On presentation, she was hemodynamically stable and her peak temperature was 38.9 °C (101.5 °F). Physical examination revealed bulky axillary lymphadenopathy (Figure 1). 

Laboratory findings revealed pancytopenia with a WBC count of 2.08 x 10^3^/uL, Hb of 6.8 g, platelet of 9 x 10^3^/uL, ANC of 1.05 x 10^3^/uL, lactic acid of 4.9 mmol/L, and glucose of 57 mg/dL (Table 1). Blood transfusions were given per hospital protocol.

**Table 1:** Lab results ANC: absolute neutrophil count; Hb: hemoglobin; WBC: white blood cells

Variables	Day 1	Day 6	Normal range
WBC, x10^3^/uL	2.1	8.2	4.4-10.5
ANC, x10^3^/uL	1.05	0.97	1.5-7.5
Hb, g/dL	6.7	6.3	11.4-14.7
Platelets, x10^3^/uL	9	8	139-361
Lactic acid	4.9	14.4	0.5-1.9 mmol/L

**Figure 2:**
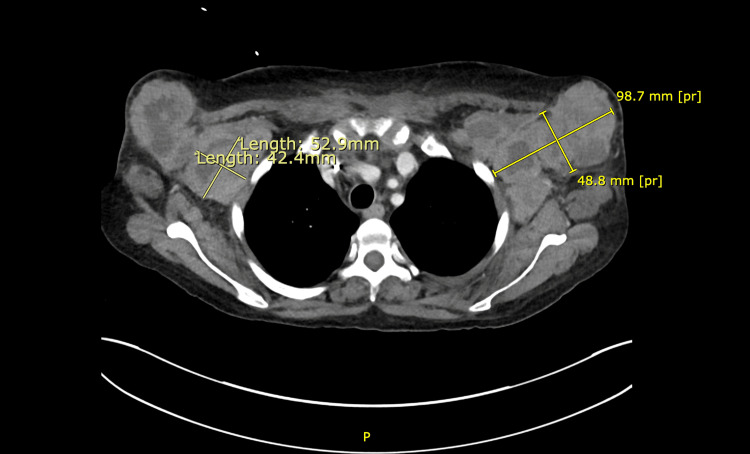
CT imaging of bilateral axillary lymphadenopathy CT: computed tomography

The patient was empirically started on intravenous linezolid and cefepime, which was changed to piperacillin-tazobactam for neutropenic fever. She was also started on micafungin and acyclovir for prophylaxis. Extensive infectious workup including chest X-ray, blood culture, and urine culture was negative. A whole-body CT scan revealed bilateral axillary lymphadenopathy with high tumor burden but did not reveal any sign of pneumonia or abscess (Figure 2).

**Figure 3:**
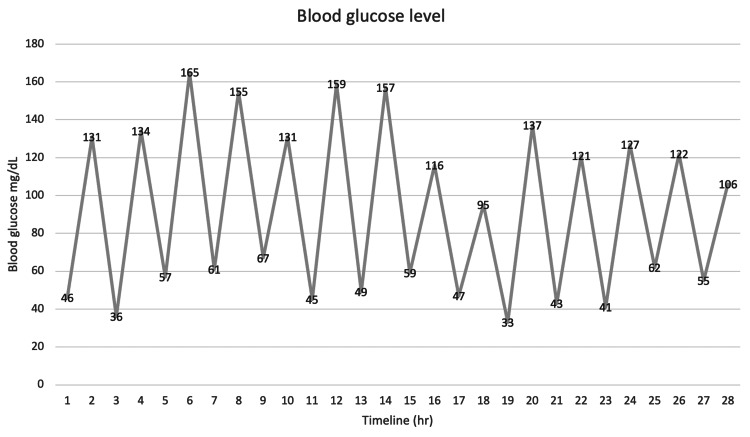
Blood glucose trend Dextrose 50 IV was given at the following timeline (hr): 1, 3, 5, 7, 9, 11, 13, 15, 17, 19, 21, 23, 25, 27

Interestingly, the patient developed resistant hypoglycemia and worsening lactic acidosis even though there was no clear evidence supporting active infection or septic shock; despite being on a regular diet and an infusion of dextrose 10%, she continued to experience drops in blood glucose levels into the 40s six times after receiving repetitive pushes of dextrose 50% (Figure 3).

The patient had no known history of diabetes, and extensive workup for endocrinologic causes of hypoglycemia including insulin, proinsulin level, C peptide, and sulfonylureas was normal; her morning cortisol level was 16.8 ug/dL (6-18.4 ug/dL). Meanwhile, her lactic acid level increased from 4.9 to 12.6 mmol/L. She was transferred to the ICU due to worsening lactic acidosis and refractory hypoglycemia. Her lactic acid peaked at 14.40 mmol/L and her blood glucose continued to drop even though she was started on dextrose 20 infusion at 150 ml/hour in the ICU. Her clinical status rapidly deteriorated and she was intubated for hemoptysis and respiratory distress; her condition was complicated by refractory shock and life-threatening bleeding due to DIC. Considering the extremely poor prognosis, the decision was made by the family to withdraw care, and the patient expired the same night after the withdrawal of care.

## Discussion

The Warburg effect is considered an oncologic emergency that requires immediate attention and intervention. It can be seen in hematological malignancy due to high tumor burden and cancer aggressiveness, however, it is occasionally reported in various types of solid tumors [3]. The Warburg effect is the underlying pathophysiological mechanism that leads to the clinical syndrome featuring type B lactic acidosis and refractory hypoglycemia in cancer patients, after ruling out sepsis or other etiologies that can explain the constellation of manifestations. In comparison to type A lactic acidosis, which is associated with tissue hypoperfusion, type B lactic acidosis driven by the Warburg effect results from the massive glycolysis turning pyruvate acid into lactic acid that exceeds the clearance capability of the liver [4]. It is important to maintain a high index of suspicion for clinical syndrome associated with the Warburg effect as the mortality associated with it can be as high as 90% [5]. 

**Figure 4:**
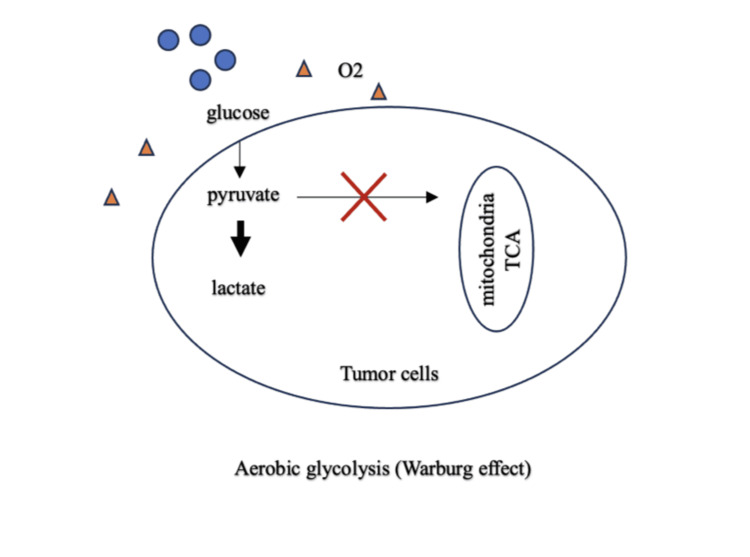
Illustration of the Warburg effect TCA: tricarboxylic acid cycle

The Warburg effect is a complex metabolic process where glucose is converted to lactate instead of being used for oxidative respiration even with the presence of oxygen (Figure 4).

Although lactate fermentation is less efficient in producing energy, it is much faster, and hence the amount of ATP generation in a given time period is comparable between these two pathways. Studies have suggested that lactate serves as a potent signaling molecule in tumor angiogenesis, migration, and immune escapes, playing an important role in the self-sufficiency of tumor cells [6]. 

The optimal treatment for patients who develop clinical syndrome driven by the underlying Warburg effect is unknown due to the rarity of this disorder and the lack of randomized controlled trials. Historically, sodium bicarbonate and hemodialysis were used for bridging to disease-modifying treatments such as chemotherapy [7]; however, there is no high-quality evidence to prove their efficacy [8,9]. Since thiamine is known as an enzymatic cofactor of aerobic oxidative phosphorylation, thiamine deficiency is considered one of the causes of type B lactic acidosis and was used in correcting the clinical syndrome driven by the Warburg effect [10]. One study has reported thiamine deficiency developing in patients with leukemia where quickly proliferating blast cells consumed large amounts of thiamine and lactic acidosis improved after thiamine replacement [11]. So far, the only outcome-modifying treatment is chemotherapy, which should be initiated promptly once the clinical syndrome associated with the Warburg effect is suspected [12]. Patients who survived the Warburg effect were started on chemotherapy three days on average before elevated lactic acid levels were first detected [13,14]. 

However, new insights into the understanding of the Warburg effect microenvironment have opened new avenues for cancer treatments [15]. Given that aerobic glycolysis is the hallmark of tumorigenesis, novel cancer therapies targeting lactate production and shuttling are under investigation. Pyruvate dehydrogenase (PDH) agonists such as dichloroacetate were able to shift the energy metabolism from lactate fermentation to the tricarboxylic acid cycle, thereby showing the potential of inhibiting tumor cell growth [16,17]. Other reports have described using dichloroacetate successfully in treating patients with non-Hodgkin lymphoma (NHL) [18]. Other therapeutic targets such as monocarboxylate transporters (MCT) inhibitors have been shown to work by limiting the transcellular transportation of lactate [19] and thereby mitigating the tumorigenic effect of lactate.

## Conclusions

The Warburg effect can be an early sign of impending clinical decompensation; early recognition enables the clinician to respond effectively to the grim nature of the illness and initiate prompt chemotherapy to improve the outcome. As a hallmark of tumor cells, the Warburg effect has also become a promising therapeutic target in cancer treatment.
